# A Hybrid Residual Attention Convolutional Neural Network for Compressed Sensing Magnetic Resonance Image Reconstruction

**DOI:** 10.3390/diagnostics13071306

**Published:** 2023-03-30

**Authors:** Md. Biddut Hossain, Ki-Chul Kwon, Rupali Kiran Shinde, Shariar Md Imtiaz, Nam Kim

**Affiliations:** School of Information and Communication Engineering, Chungbuk National University, Cheongju-Si 28644, Chungcheongbuk-Do, Republic of Korea

**Keywords:** MRI reconstruction, attention mechanism, compressed sensing, data augmentation, deep learning, dual residual CNN

## Abstract

We propose a dual-domain deep learning technique for accelerating compressed sensing magnetic resonance image reconstruction. An advanced convolutional neural network with residual connectivity and an attention mechanism was developed for frequency and image domains. First, the sensor domain subnetwork estimates the unmeasured frequencies of k-space to reduce aliasing artifacts. Second, the image domain subnetwork performs a pixel-wise operation to remove blur and noisy artifacts. The skip connections efficiently concatenate the feature maps to alleviate the vanishing gradient problem. An attention gate in each decoder layer enhances network generalizability and speeds up image reconstruction by eliminating irrelevant activations. The proposed technique reconstructs real-valued clinical images from sparsely sampled k-spaces that are identical to the reference images. The performance of this novel approach was compared with state-of-the-art direct mapping, single-domain, and multi-domain methods. With acceleration factors (AFs) of 4 and 5, our method improved the mean peak signal-to-noise ratio (PSNR) to 8.67 and 9.23, respectively, compared with the single-domain Unet model; similarly, our approach increased the average PSNR to 3.72 and 4.61, respectively, compared with the multi-domain W-net. Remarkably, using an AF of 6, it enhanced the PSNR by 9.87 ± 1.55 and 6.60 ± 0.38 compared with Unet and W-net, respectively.

## 1. Introduction

Magnetic resonance imaging (MRI) is a noninvasive and sophisticated medical imaging method that sheds light on the anatomical structure and operation of the human body and brain [1] by generating high-quality images. Each tissue’s unique properties can be recognized using a novel MRI acquisition method. Based on the different tissue signal diversities, MRI reconstructs quantitative images that are very important for the early diagnosis of sickness or physical changes. MRI does not entail exposure to harmful radiation [2], unlike X-rays, photoacoustic tomography, and computed tomography. However, the long image acquisition time [3] makes it challenging to use MRI in time-sensitive situations, such as in cases of stroke, although acquisition time can be reduced given that MRI systems allow for comprehensive control of data acquisition. Acquired frequencies in MRI are stored in k-space instead of image space. K-space is a matrix the same size as the reconstructed image that stores complex (real and imaginary) raw MRI data. Every point in this matrix holds a portion of the data needed to create the entire image. The periphery of k-space possesses high spatial frequency that depicts information concerning image edges, details, and sharp transitions. On the other hand, the central area of k-space retains the high spatial frequency that expresses the image at its brightest. Fully sampled k-space is essential for obtaining high-resolution images but increases acquisition time. Acquiring a few frequencies is one of the most popular methodologies for rapid MRI reconstruction. However, due to undersampling, tissue structures are often distorted, and aliasing artifacts appear in the images. Compressed sensing (CS) [4] randomly employs an iterative process to select appropriate frequencies for reconstructing suitable images from sparsely sampled MRI data. However, these iterative methods are time-consuming, which makes them challenging to use in conjunction with fast MRI.

Deep learning (DL) has been effectively applied for the analysis of medical images [5,6]. Deep neural networks have emerged in medical image reconstruction, classification, and computer-based disease identification [7]. The early detection of a tumor is more important for effective treatment, especially when seeking to avoid surgery and reduce the risk of death. DL in computer-aided diagnosis (CAD) systems increases the accuracy and efficiency of diagnosing the normal (non-tumor zone) and abnormal (tumor zone) tissues at different stages that can be used in smart healthcare systems. Recently, DL has been used to address the shortcomings of iterative conventional CS techniques. DL is crucial for the efficient generation of high-quality images from undersampled k-spaces and obviates the need for repeated processing after the model has been appropriately trained by providing it with both fully and partially sampled k-spaces from corresponding images. A neural network [8] is first utilized to lessen aliasing artifacts in multi-coil MRI [9]. DL-based repetitive unrolled optimization approaches [10,11,12] can then be applied for CS-MRI reconstruction to better learn image features; however, the processing times for the iteration are relatively long and the ill-posed inverse problem remains to be solved. Several strategies [13,14,15,16,17,18,19,20,21,22] have been used to enhance the quality of distorted images. Distorted images can be reconstructed from sparse spatial frequencies using an inverse fast Fourier transform (IFFT); however, the visual characteristics may be restored incorrectly when frequencies are significantly sparse. Frequency domain networks [23,24,25,26] estimate the unknown missing k-space frequencies before image conversion; these networks also tend to recover low-importance sensor data that increase their reconstruction time. Direct mapping networks [27,28] straightforwardly convert the frequency into an image; however, they only work for relatively small images (<128 × 128 pixels) due to the large memory requirements. Single-domain networks are usually applied to the sensor or image domain, but the resulting reconstructed images contain significant artifacts that obscure the image’s content.

**Figure 1:**
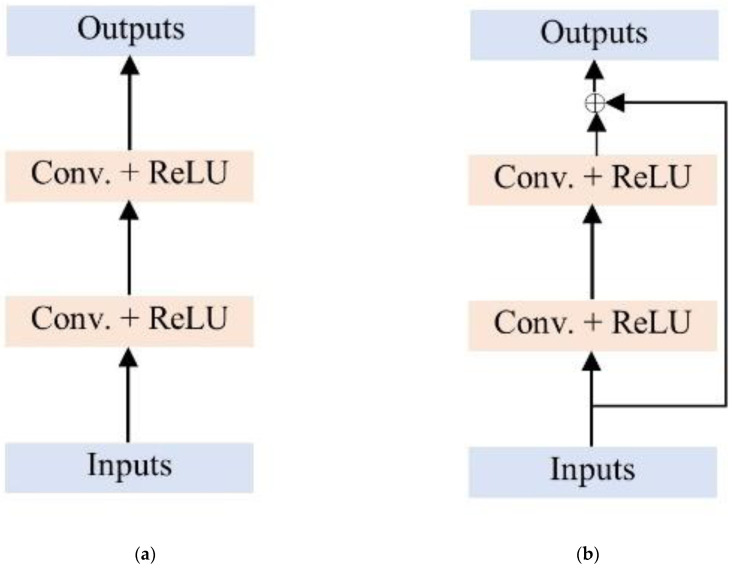
The fundamental structure of the convolutional neural network (CNN): (**a**) traditional CNN and (**b**) residual CNN.

Dual-domain methods [29,30,31,32,33] work in both the frequency and image domains by connecting inverse or direct Fourier transforms (IFT and FT, respectively) and benefit from the complementary characteristics of the two spaces. The performance of some cross-domain approaches has surpassed that of deep cascade convolutional neural networks (DC-CNNs) [34]. KIKI-net [29] deploys four CNNs sequentially for iteration, whereas a hybrid cascade [30] uses six cascade CNNs with six IFT/FT blocks. A hybrid W-net [35] combines a Unet [36] for the image domain with a residual Unet for the k-space through an IFFT; however, this model lacks data consistency measures. The use of a residual CNN in the sensor domain and a dense CNN in the image domain has been demonstrated [31]. IKWI-net [32] applies a CNN to zero-filled images first, as opposed to raw MRI data. The dual-task approach [33] uses two data consistency blocks with IFT and FT operations and performs unsupervised reconstruction using two Unet blocks. State-of-the-art multi-domain networks commonly use either Unet or residual Unet architectures. However, due to the multiple FT/IFT blocks with cascade CNNs, training and testing times must be adjusted for different sampling rates. Moreover, these multi-domain methods split the real and imaginary components into two channels instead of using complex-valued networks. This procedure does not preserve the data’s phase information. Thus, the development of complex-valued networks is essential for quantitative and qualitative images. A comparison of non-residual and residual CNNs for MRI reconstruction was conducted [37]; the findings have since been incorporated into more recent models [38]. A fundamental block diagram of non-residual and residual CNNs is shown in Figure 1. Traditional CNNs have a severe flaw in that they must learn the total feature map, which requires a large number of parameters. As a result, they are costly to train and slow to run. Residual networks (ResNets) are a type of neural network developed as an enhancement over regular CNNs. It is a form of CNN in which the previous layer’s input is added to the current layer’s output. This skip connection facilitates network learning and leads to improved performance. The ResNet architecture has demonstrated performance in various tasks, including image classification, object detection, and semantic segmentation.

Here, we introduce an advanced dual-domain MRI reconstruction approach that uses a complex-valued residual attention convolutional neural network (RA-CNN) to modify the multi-domain W-net architecture through residual connectivity and attention mechanisms for frequency and image domains. Recently, the attention mechanism has been effectively used for computer-based medical diagnosis. CNNs can easily incorporate this mechanism to automatically highlight salient elements. The proposed complex-valued RA-CNN is comprised of two subnetworks: one each for the sensor and image domains. First, the sensor domain subnetwork predicts the unmeasured frequencies of k-space to reduce aliasing artifacts. Second, the image domain subnetwork performs a pixel-wise operation to remove blur and noise artifacts. Skip connections efficiently concatenate the feature maps to alleviate the vanishing gradient problem that occurs as network depth increases, thus preventing interruption of the network training procedure. Integration and communication between the two subnetworks promotes data consistency and more efficient learning of the features from both domains. At the same time, the attention gate (AG) in each decoder layer enhances network generalizability and speeds up image reconstruction by eliminating irrelevant activations, without an iteration process. Therefore, the proposed technique reconstructs real-valued clinical images from sparsely sampled k-space that are identical to the fully sampled k-space images. These images appear to be of higher quality than those produced using other dual-domain cascade networks or single-domain methods. Complex-valued data augmentation (DA) is also applied to overcome data scarcity issues.

## 2. Proposed Methodology

**Figure 2:**
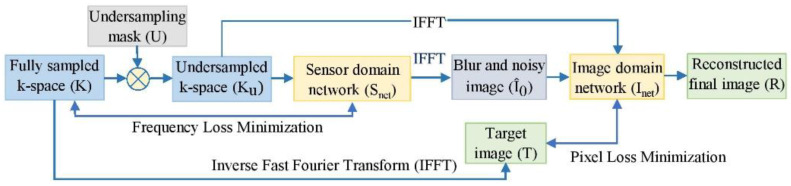
Flowchart of the proposed dual-domain magnetic resonance imaging (MRI) reconstruction technique.

According to the Nyquist–Shannon theorem [39], discrete Fourier methods can only access information when the sampling rate is double the bandwidth of the recorded continuous-time signal. MRI relies heavily on this Nyquist rate. If pertinent preceding information is acquirable, reconstruction techniques other than Fourier analysis can be used to retrieve valuable information from sparsely sampled data below this rate. Hence, we developed an RA-CNN technique that calculates intrinsic correlations between fully sampled and undersampled k-spaces, along with their reconstructions. The RA-CNN consists of two neural subnetworks: sensor domain and image domain networks. These subnetworks are trained from end to end through sparsely sampled complex-valued MRI data. The skip connections of residual CNNs perform better than traditional CNNs and execute much faster. Added novel attention gates with each skip connection help to extract the more important features from both the signals and images. The k-spaces and images reconstructed by these subnetworks are similar to fully sampled k-spaces and images. Figure 2 shows the workflow of our proposed multi-domain MRI reconstruction method, i.e., the RA-CNN, which uses two RA Unet blocks connected by an IFFT.

The undersampled k-space ($K_{u}$) is generated by elementwise multiplication between the entire k-space (K) and the sub-sampling mask (U). First, the sensor/frequency network $S_{\mathrm{net}}$ tries to estimate the unmeasured frequencies by reducing frequency loss between the fully sampled and reconstructed k-space. The initial image ${\hat{I}}_{0}$ is then reconstructed by IFFT from the output of the first network. Finally, blurring and noise artifacts are removed by the image/spatial domain network $I_{\mathrm{net}}$, which is achieved by reducing pixel disparity between the reconstructed final output (R) and the fully sampled reference image (T).

### 2.1. Deep Learning

DL methods accelerate MRI reconstruction and improve image quality through the signal projection of undefined regions. This interpolation removes aliasing artifacts by satisfying the Nyquist rate. Here, a DL model reconstructs unique suitable images by fitting the sparsely sampled data. Notably, our approach does not generalize the signal to indeterminable regions, unlike conventional band-limited signal extrapolation techniques [40] and low-rank modeling of local k-space neighborhoods [41].

The proposed method is described below in terms of the sensor domain network, IFFT operation, and image domain network.

#### 2.1.1. Sensor Domain Network

The sensor domain network, $S_{\mathrm{net}}$, attempts to fully regain the k-space, ${\hat{K}}_{\mathrm{norm}}$, from the undersampled k-space, $K_{u}$. Mathematically, this is represented as

(1) $${\hat{K}}_{\mathrm{norm}}=S_{\mathrm{net}}{{(K}_{u}}_{\mathrm{norm}})$$

where $K_{u}{}_{\mathrm{norm}}$ represents the normalized undersampled k-space and is expressed by

(2) $$K_{u}{}_{\mathrm{norm}}=\frac{K_{u}{-\;\mu K}_{u}{}_{\mathrm{train}}}{{\sigma K}_{u}{}_{\mathrm{train}}}$$

where ${\mu K}_{u}{}_{\mathrm{train}}$ and ${\sigma K}_{u}{}_{\mathrm{train}}$ represent the mean and standard deviation (SD) of the given undersampled k-spaces, respectively. This network uses complex, normalized, two-channel (real and imaginary), and sparsely sampled raw MRI data as input and outputs the complex k-space.

#### 2.1.2. Inverse Fast Fourier Transform

The reconstructed k-space of the sensor domain network is denormalized before IFFT. The normalization can be reversed by

(3) $$\hat{K}={K_{u}}_{\mathrm{norm}}\;\times \;\sigma {K_{u}}_{\mathrm{train}}+\mu {K_{u}}_{\mathrm{train}}$$

After denormalization of the reconstructed k-space to yield $\hat{K}$, it is transformed into images using the IFFT ($F^{-1}$) operation:

(4) $${\hat{I}}_{0}=F^{-1}(\hat{K})$$

where ${\hat{I}}_{0}$ indicates the initial reconstructed image. There are no trainable parameters for this section.

#### 2.1.3. Image Domain Network

The image domain network $I_{\mathrm{net}}$ takes the abovementioned initial reconstructed image as input, which is renormalized to increase the convergence speed of the network:

(5) $${{\hat{I}}_{0}}_{\mathrm{norm}}=\frac{{\hat{I}}_{0}-{\mu I_{0}}_{\mathrm{train}}}{\sigma {I_{0}}_{\mathrm{train}}}$$

where ${\mu I}_{0}{}_{\mathrm{train}}$ and ${\sigma I}_{0}{}_{\mathrm{train}}$ represent the mean and SD of the elementary reconstructed image, respectively. This normalized image then traverses the network to generate the final output image, R:

(6) $$R=I_{\mathrm{net}}({{\hat{I}}_{0}}_{\mathrm{norm}})$$

This network also uses a residual attention Unet architecture through concatenation with the undersampled input k-space.

## 3. Proposed Network Architecture

Our advanced RA-CNN was designed based on residual connectivity and modification of the AG [42]. Batch normalization [43] speeds up the training process compared with the baseline Unet. Unet possesses 23 layers, whereas the RA-CNN possesses 81 convolutional and deconvolutional layers. The gradient details must pass across many tiers and could dissipate before they reach subsequent layers, which causes the vanishing gradient problem. Residual connectivity lessens the likelihood of a vanishing gradient and simplifies the network train.

**Figure 3:**
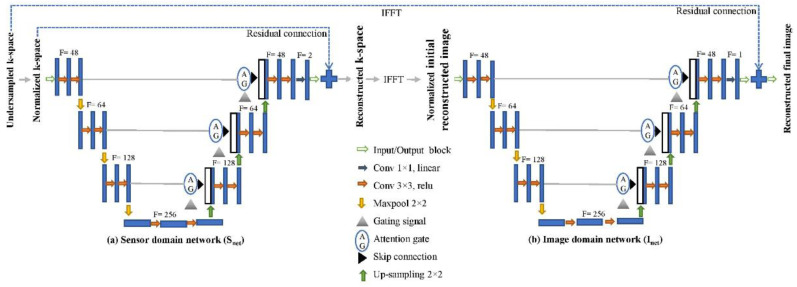
Proposed dual-domain residual attention convolutional neural network (RA-CNN) architecture.

The sensor domain network structure shown in Figure 3 (left side) consists of two main subdivisions: the down-sampling and up-sampling sections. The down-sampling section consists of four consecutive convolutional blocks (CBs) used for extracting k-space features. Every CB contains two 3 × 3 convolutional layers with a rectified linear unit (ReLU) [44] activation function and padding = 1. The first CB is applied to the 256 × 256 normalized undersampled k-space with 48 kernels. The channel numbers then gradually increase by 64, 128, and 256. After each CB, with the exception of the last CB in the decoding section, a max-pooling operation is performed. At each step, this process doubles the feature number and halves the input dimension. Up-sampling involves 2 × 2 deconvolution (upscaling), an AG, and CBs. The up-sampling portion re-establishes the size of the features and preserves the symmetric form of the encoding portion. The loss of data generated by the decoding/encoding operation is reduced by this balanced form, which also allows for the reprocessing of features through their concatenating in the associated layer. The features of both layers flow through the AG before concatenation. The AG can determine the correlations between frequencies and create long dependencies to access important information. The last layer of this section executes a linear 1 × 1 convolutional operation using two filters. Finally, this network generates complex k-space by concatenating the last layer’s output and normalizing the undersampled k-space through a residual connection.

The image domain network structure shown in Figure 3 (right side) also has two main sections: the down-sampling and up-sampling sections. In the down-sampling section, the first CB is applied to the 256 × 256 normalized initial reconstructed images with 48 filters. The filter numbers then gradually increase by 64, 128, and 256. After each CB, a max-pooling process is executed to extract more specific image features, with the exception of the last CB in the decoding section. The up-sampling step, in contrast, retains the symmetric shape of the encoding block and preserves the dimensions of the feature maps. At every skip connection, the features of upper and lower layers flow via the AG. The AG assembles the essential features of various types of spatial information. The last layer of this part performs a linear 1 × 1 convolutional operation with a single filter. The final image is reconstructed by concatenating the output of the last layer of the network and inputting the undersampled k-space.

### Attention Gate

The attention mechanism [45] for medical image analysis automatically accrues new information by focusing on target structures of varying size and shape. Models with AGs intuitively discover the important hidden elements from an input image for a certain task. To increase model sensitivity and prediction accuracy, AGs may be readily attached to popular CNNs such as Unet, without any increase in computing complexity. In an encoder–decoder-based approach, various low-level feature extractions are carried out during feature interpretation in the first few layers. The redundant features are reduced through active suppression using AGs at the skip connections. Two inputs, g and x, are required for every AG. The next bottom layer of the network provides the gating signal, g; since it comes from a more extensive area of the network, it accurately represents further useful features. The input feature, x, is the outcome of skipped connections that arise from the early phases and, notably, provides better spatial information.

**Figure 4:**
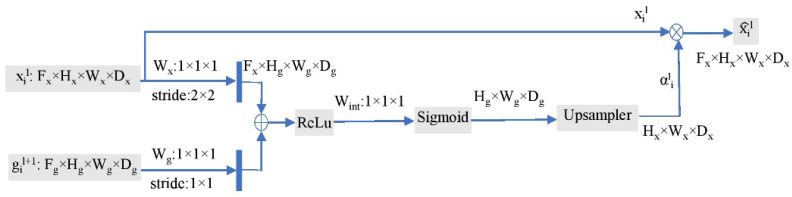
The attention gate schematic diagram.

As presented in Figure 4, input features $x_{i}^{l}$ execute a 1 × 1 convolution operation with a stride of 2 × 2 to decrease the size (H × W) by half, whereas gating signals $g_{i}^{l+1}$ execute a 1 × 1 convolution operation with a stride of 1 × 1. Consequently, the gating signals and updated input features maintain the same spatial geometry. Using elementwise summation, the ReLU activates them before mapping by $W_{\mathrm{int}}^{T}$ into a lower-dimensional space for gating procedures. The vector in [0, 1] is leveled by the sigmoid function, with coefficients closer to 1 indicating more important traits. The dimension of the attention weighting matrix $\alpha_{i}^{l}$ is then restored to match the pixel intensity of the provided input features using a trilinear up-sampler. The attention weighting matrix $\alpha_{i}^{l}$ and input features $x_{i}^{l}$ are multiplied elementwise to produce the output ${\hat{x}}_{i}^{l}$ of the AG, which is then sent to the regular CBs.

## 4. Experimental Details

### 4.1. Dataset and Undersampling Mask

In this simulation, we used publicly available Calgary–Campinas [46,47] T1-weighted, fully sampled brain MRI k-space data. The dataset was acquired using a magnetic resonance scanner (Discovery MR750; General Electric Healthcare, Waukesha, WI, USA) via a collaboration between researchers at the University of Calgary’s Vascular Imaging Lab (Calgary, AB, Canada) and the Medical Image Computing Lab at the University of Campinas (São Paulo, Brazil). A total of 45 k-spaces were used, where every k-space had 170 sagittal cross-sectional T1-weighted MRI sequence images (256 × 256). In total, 25 k-spaces (4250 slices) were used during training, 10 k-spaces (1700 slices) were used for validation, and another 10 k-spaces (1700 slices) were used only for testing. In the training, validation, and testing phases, there were no repeat object slices. During training, the zero-filled undersampled k-space was used for network input. The fully sampled k-space and corresponding fully sampled images were used as the network target outputs. To train and test each network, two-dimensional Gaussian undersampling sequences were employed. Simulation analyses were conducted using four-, five-, and six-fold acceleration factors (AFs).

#### Data Augmentation

DL models require large amounts of training data to avoid overfitting and underfitting issues. However, it can be expensive and time-consuming to gather well-annotated medical data. DA [48] is frequently used in DL to enhance the size and heterogeneity of the training set and thus significantly improve efficiency while decreasing generalization errors in DL-based models. DA strategies are less popular for image reconstruction applications and are also considerably more challenging due to the extensive measurements (i.e., complex data) involved [49]. By providing fully sampled complex MRI scans, augmented training data can be generated that include the undersampled k-space along with reference images and the associated k-space. The following augmentation processes were applied using the proposed RA-CNN method:

- Rotation range = 40 degrees;
- Height shift range = 0.075%;
- Width shift range = 0.075%;
- Zoom range = 0.25%;
- Shear range = 0.25%;
- Vertical flip = true;
- Horizontal flip = true;
- Fill mode = nearest.

**Figure 5:**
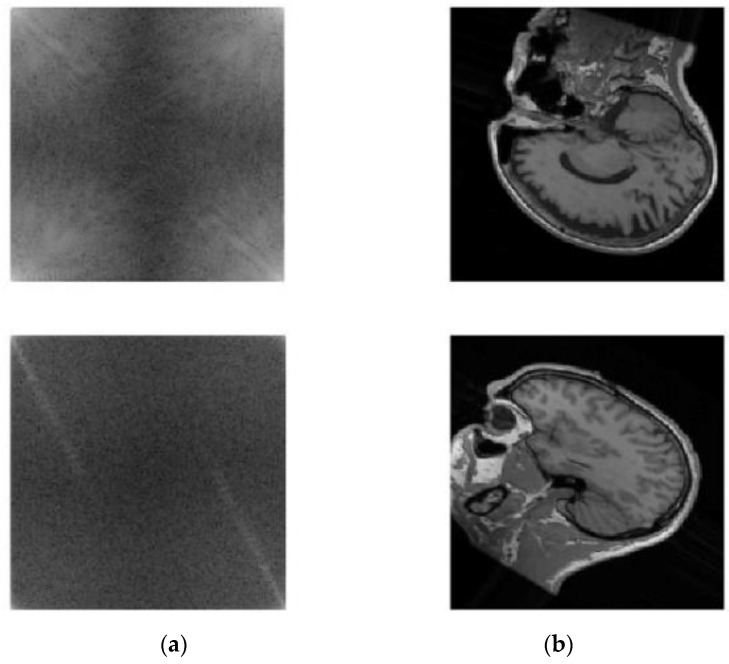
Data augmentation: (**a**) augmented k-space and (**b**) the corresponding reconstructed image.

The visual effects of the augmented k-spaces and their corresponding images are shown in Figure 5. Notably, the field strengths of the MRI scanners used for acquisition may have differed. DA impacts the ability to generalize new MRI scanner models (i.e., those not available during training). On unknown scanner sequences, DA can enhance reconstruction quality.

### 4.2. Evaluation Metrics

Network performance was evaluated using three parameters: the structural similarity index measure (SSIM) [50], normalized root mean square error (NRMSE), and peak signal-to-noise ratio (PSNR). The SSIM perceptual index measures the similarity between two images by comparing the reciprocal dependencies between neighboring pixels with respect to contrast, structural characteristics, and brightness. The SSIM between the desired image (T) and reconstructed image (R) is given by

(7) $$\mathrm{SSIM}\;\left(T,\;R\right)\;=\;\frac{\left({2\mu }_{T}\mu_{R}{+c}_{1}\right)\left({2\sigma }_{\mathrm{TR}}{+c}_{1}\right)}{\left(\mu_{T}^{2}{+\mu }_{R}^{2}{+c}_{1}\right)\left(\sigma_{T}^{2}{+\sigma }_{R}^{2}{+c}_{1}\right)}$$

where $\mu_{T}$ and $\mu_{R}$ are the average values of T and R, respectively, $\sigma_{T}^{2}$ and $\sigma_{R}^{2}$ represent the respective pixel variance values, and $\sigma_{\mathrm{TR}}$ is the covariance value. $c_{1}$ and $c_{2}$ are calculated to assist with data division:

$$c_{1}\;=\;{(0.01b)}^{2}{,\;c}_{2}\;=\;{(0.03b)}^{2}\;\mathrm{with}\;b\;=\;\max\left(T\right)–\min\left(T\right)$$

NRMSE compares pixel disparities between the network predictions and reference values, which is calculated as follows:

(8) $$\mathrm{NRMSE}\left(T,\;R\right)\;=\;\frac{\sqrt{\frac{1}{N}\sum_{i\;=\;1}^{N}{{(T}_{i}{–R}_{i})}^{2}}}{\max\left(T\right)–\min\left(R\right)}$$

where $T_{i}$ and $R_{i}$ represent the fully sampled and reconstructed images, respectively, and N represents the size of the images. PSNR measures the relationship between the signal’s peak potential power and the noise that degrades fidelity:

(9) $$\mathrm{PSNR}\left(T,\;R\right)\;=\;{20\log}_{10}\left(\frac{255}{\sqrt{\frac{1}{N}\sum_{i\;=\;1}^{N}{{(T}_{i}{–R}_{i})}^{2}}}\right)$$

These metrics were chosen because they are frequently applied for image reconstruction assessment. Higher SSIM and PSNR values denote a better outcome. On the other hand, smaller NRMSE values indicate better images.

### 4.3. Loss Function

The loss function assesses the difference between the fully sampled and sub-sampled images. The main objective of the proposed method is to reduce the value of this loss function, in which a smaller difference between the fully sampled and undersampled images indicates efficient reconstruction. Reconstruction performance can be enhanced using an appropriate loss function that provides precise gradient data for network training. The weighted sum of the NRMSE in each domain was used as the loss function to compute frequency and pixel-wise disparities and is expressed as

(10) $$\mathrm{Loss}=\frac{1}{M}\sum_{i=1}^{M}\{\omega \;\times \;\mathrm{NMRSE}\;(K_{i},\;{\hat{K}}_{i})\;{+\left(1-\omega \right)\;\times \;\mathrm{NRMSE}\;(T}_{i},\;R_{i})\}$$

where $K_{i}$ and ${\hat{K}}_{i}$ represent the target and reconstructed k-spaces in the sensor domain, respectively; $T_{i}$ and $R_{i}$ denote the target and reconstructed images in the spatial domain of the i-th instance in the training dataset, respectively; and M is the number of training instances. With our method, the initial weight (ω) was 0.001; this value was updated continuously to minimize loss.

### 4.4. Experimental Setup

**Figure 6:**
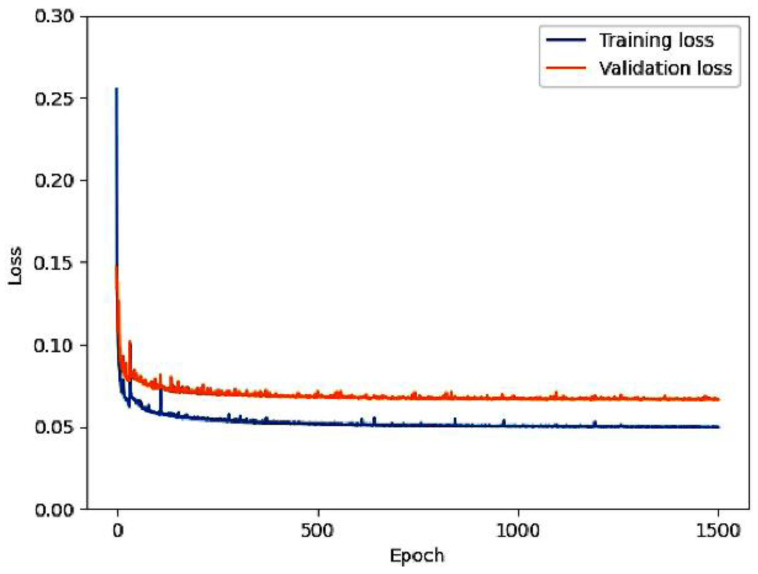
Training and validation losses of the proposed technique.

A Windows 10 Pro 64-bit computer with an Intel core i7-9800X 3.80 GHz processor, 128 GB of RAM, and an NVIDIA GeForce RTX 2080 Ti graphics processing unit was used for model training and testing. The TensorFlow v2.4 and Keras v2.4 open-source libraries were utilized to implement the models in Python 3.8 and PyCharm environments. In the RA-CNN, the loss function was reduced by an Adam optimizer using the momentum of β1 = 0.9 and β2 = 0.999. The initial learning rate was $10^{-3}$ but was subsequently reduced to $10^{-7}$. A small batch size (*n* = 16) was used, and 1500 epochs were employed for training the proposed RA-CNN. The proposed network’s training and validation losses are illustrated in Figure 6; the measurements show that the normalizing effect of the residual connection reduces the probability of overfitting.

## 5. Results and Discussion

The effectiveness of our proposed RA-CNN was compared with direct mapping and single- and multi-domain networks. Unet [36], the de-aliasing generative adversarial network (DAGAN) [13], RefineGAN [14], the projection-based cascade Unet (PBCU) [51], and the fully dense attention (FDA)-CNN [52] are single-domain techniques, while the DC-CNN [34], KIKI-net [29], W-net [35], the hybrid cascade [30], and the dual-encoder Unet [53] are multi-domain approaches. The GAN-based DAGAN applies a residual Unet architecture for the generator and combined adversarial and innovative content losses. RefineGAN measures cyclic loss using a residual Wasserstein GAN [54]. PBCU uses five consecutive Unet blocks, and the FDA-CNN employs attention mechanisms with a densely connected CNN. The DC-CNN utilizes multiple cascading CNNs for MRI image enhancement. KIKI-net uses four Unet blocks, which sequentially operate in k-space, image space, k-space, and image space. W-net uses two Unet blocks, and the hybrid cascade uses six Unet blocks. The dual-encoder Unet applies decomposing automated transform by manifold approximation (dAUTOMAP) [28] and two encoders within the Unet framework. The implementation and hyperparameters were based on original research for each approach. Simulations were conducted to test the performance of the proposed RA-CNN when using the abovementioned state-of-the-art techniques; SSIM, NRMSE, and PSNR values were evaluated through numerical analysis as performance metrics.

**Figure 7:**
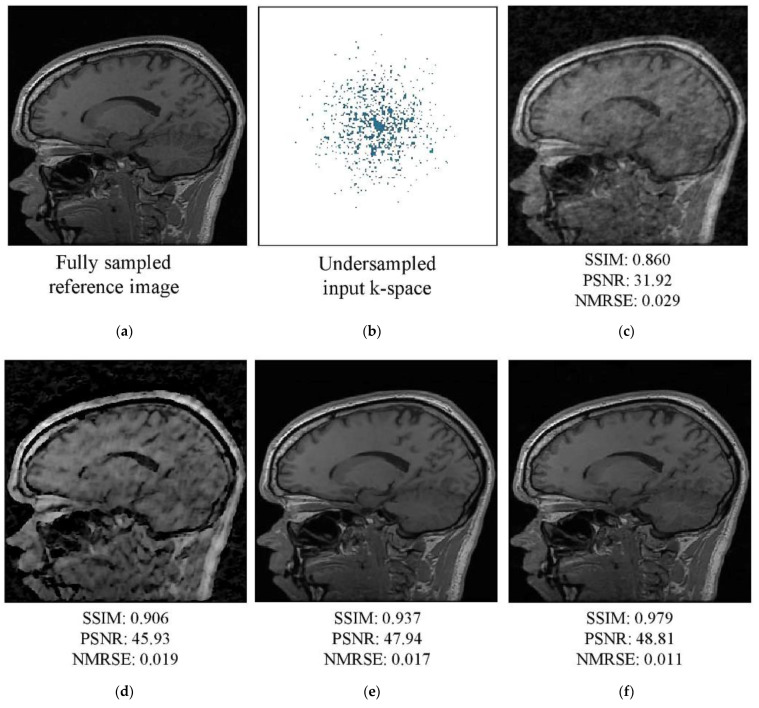
MRI images from the test dataset (slice No. 100) for acceleration factor (AF) 4: (**a**) fully sampled reference image and (**b**) undersampled k-space. Image reconstructed by the (**c**) inverse fast Fourier transform (IFFT), (**d**) Unet, (**e**) W-net, and (**f**) RA-CNN methods.

**Figure 8:**
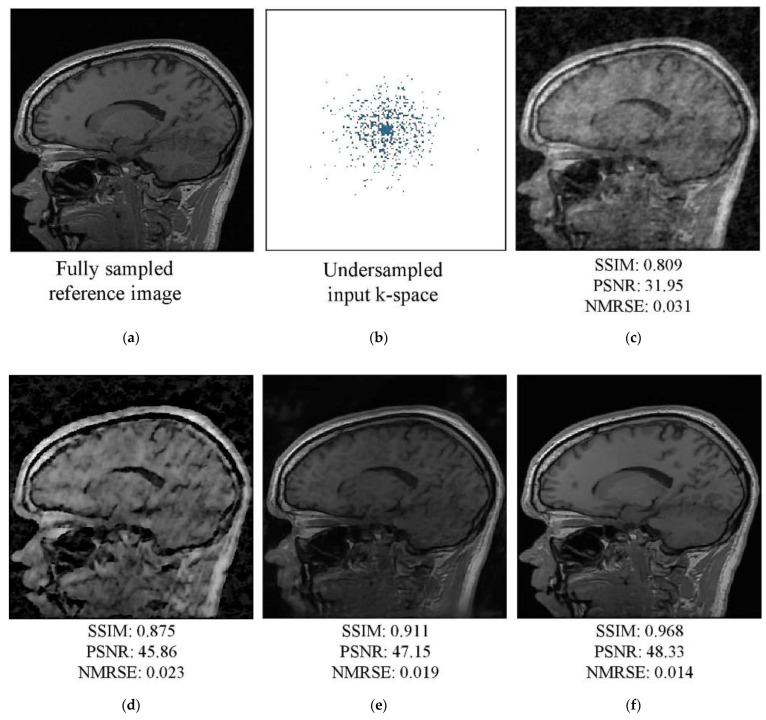
MRI images from the test dataset (slice No. 100) for AF 5: (**a**) fully sampled reference image and (**b**) undersampled k-space. Image reconstructed by the (**c**) IFFT, (**d**) Unet, (**e**) W-net, and (**f**) RA-CNN methods.

An AF of 4 was used to train the networks, which were evaluated using four-, five-, and six-fold AFs (AFs 4–6, respectively). Visual assessment of the tested k-spaces and reconstruction images for AFs 4 and 5 are shown in Figure 7 and Figure 8, respectively. In the figures, the first row shows the fully sampled reference image (a), undersampled k-space (b), and reconstructed undersampled image (c). The second row shows the images reconstructed by the Unet (d), W-net (e), and RA-CNN (f) networks using the undersampled k-space. The undersampled image was generated using an IFT; the image exhibited unnatural inconsistent artifacts and blurred edges. The single-domain network Unet enhanced the qualitative values of the initial zero-filling image. The hybrid W-net performed better than Unet and improved the image quality and quantitative values. Moreover, the RA-CNN more accurately reconstructed the undersampled k-space as the target image than the single- and multi-domain networks. Specifically, the RA-CNN focused on the features essential for diagnosis during image reconstruction and performed better than the other techniques in terms of the elimination of artifacts. The RA-CNN produced superior quantitative values for a specific slice (No. 100) of the reconstructed image compared to the single- and multi-domain networks.

The mean and standard deviation SSIM, NMRSE, and PSNR values obtained using the various state-of-the-art methods for AFs 4 and 5 are shown in Table 1. Along with these quantitative results, clinical parameters such as edge sharpness, motion fidelity, artifacts, image distortion, and diagnostic score are essential for accurately diagnosing the reconstructed images. To assess statistically significant changes, we utilized one-way analysis of variance (ANOVA) and post hoc paired *t*-tests. Statistical importance was determined using a *p*-value < 0.01. The one-way ANOVA testing revealed differences (*p* < 0.01) with statistical significance among all measurements and acceleration parameters. Multi-domain networks generated more accurate quantitative results than single-image-domain networks. These observations and numerical analyses showed that the proposed RA-CNN generated the most accurate SSIM and PSNR values, although that was not the case for the NRMSE values. The hybrid cascade approach yielded better NRMSE values under both sampling rates, although the difference in performance from the RA-CNN was very small in this respect. The paired *t*-tests revealed that the RA-CNN outperformed the other approaches in these assessments.

**Figure 9:**
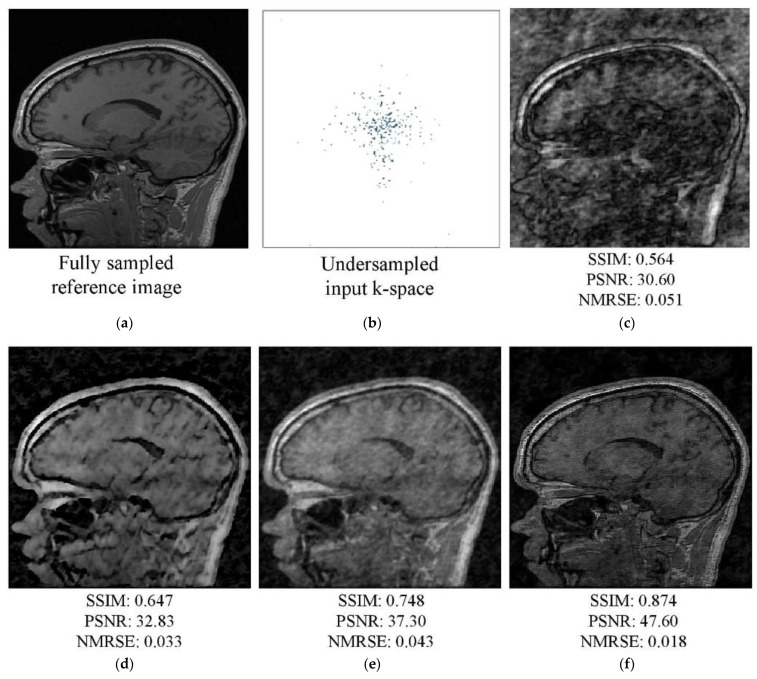
MRI images from the test dataset (slice No. 100) for AF 6: (**a**) fully sampled reference image and (**b**) undersampled k-space. Image reconstructed by the (**c**) IFFT, (**d**) Unet, (**e**) W-net, and (**f**) RA-CNN methods.

Figure 9 depicts a fully sampled slice (No. 100) (a) and its corresponding undersampled k-space (b) for AF 6. The zero-filling image (c) contains severe noise and blur artifacts. Unet (d) showed a slight improvement in qualitative value, and the hybrid W-net (e) performed better than Unet. The output image of the RA-CNN was of higher quality than the images of other models, and the details were better restored. This appropriate reconstruction with artifact-free high temporal resolution is essential for a number of medical image post-processing activities such as classification and segmentation.

The mean and standard deviation SSIM, NMRSE, and PSNR values obtained using the various state-of-the-art methods for AF 6 are shown in Table 2, along with the number of parameters for each network. According to these data, the RA-CNN outperformed all of the other networks, followed by the FDA-CNN with the AG. The cascade CNN showed higher qualitative values for the measured metrics. Compared to the standard Unet and cascade CNN models, the proposed RA-CNN improved the mean PSNR value by 9.87 and 6.11 dB, respectively. The paired *t*-tests revealed that the RA-CNN outperformed the other approaches in this acceleration factor. Direct mapping dAUTOMAP computes only 0.16 M parameters. On the other hand, the FDA-CNN and dual-encoder Unet have almost 1 M trainable parameters. Attention mechanism-based models require fewer parameters than the more sophisticated Unet-based cascade networks; specifically, Unet, Wnet, and PBCU have approximately 3.13 million (M), 1.13 M, and 3.15 M parameters, respectively, whereas the RA-CNN has 0.68 M parameters. A compromise between scan time and image quality results in an MR exploration. Optimizing an MR exploration procedure and its sequence parameters will be necessary based on the organs and disease.

The results demonstrate that the RA-CNN was superior in terms of generating aliasing artifact- and blur-free images. Our multi-domain approach uses two residual Unet architectures with AGs and maintains symmetric encoders and decoders on either side of the network. The first advantage of this framework is that it establishes long-range connections between the encoder and equivalent decoder parts, thus allowing for the merging of various pieces of hierarchical information from the encoder and decoder and thereby increasing the network’s precision and scalability. Unet, Wnet, and PBCU have 23, 43, and 74 convolutional layers, respectively, whereas the RA-CNN has 81 convolution and deconvolution layers. The vanishing gradient issue in a neural network such as the RA-CNN poses a challenge with respect to the training results obtained during backpropagation; however, the shallow residual connection solves this issue. Model performance is enhanced by the residual units, which directly convey features from the early to end stage of convolution. As demonstrated in Figure 6, the regularization effect of residual connections decreases the risk of overfitting the training data. However, if the steps used for obtaining consistent cascade data are applied, computing time and costs increase. AGs merge lower and higher spatial data to identify meaningful features during a single computation. Consequently, the RA-CNN model requires fewer parameters than the single- and dual-domain networks. The single-domain models require 0.6–1.0 s (s) for each slice reconstruction. The cascade dual-domain methods require > 1 s per image reconstruction. The proposed RA-CNN generates better images within an average of 0.6 s. Therefore, computation time and cost are reduced. Notably, the results showed that the AG-based methods performed better than the other single and cascade networks at higher AFs. Even though we are currently just testing our method with brain data and different sampling rates, not with other types of MRI datasets involving areas such as the knee or abdomen, the results are still significant.

## 6. Conclusions

The proposed dual-domain RA-CNN reconstructs MRI images from sparsely sampled k-space data using two neural networks. The first CNN in the sensor domain predicts unacquired frequencies and then applies the second CNN in the image domain for image enhancement. Furthermore, each network has a unique impact on MRI reconstruction. Edge content and geometry are restored more effectively from undersampled k-space using this multi-domain CNN. As a CNN is used directly to retrieve the sensor data, some lower frequencies might be recoverable. Consequently, this method is capable of extracting realistic visual features and reconstructing images that are identical to real images. Since the visual characteristics are preserved, radiologists can interpret data accurately and rapidly. Residual connection significantly enhances feature reuse and network data flow. Moreover, AGs mix lower and higher spatial data to identify valuable features while using fewer parameters than the other sophisticated Unet-based methods. Although network training takes a long time, images can be generated rapidly after training.

We show that the aggregation of two domains has an impact on MRI reconstruction performance. In end-to-end reconstruction based on residual and attention mechanisms, the RA-CNN performed better than several alternative single- and multi-domain networks, as reflected in the PSNR and SSIM values under various sampling rates. In future research, we will apply our strategy for interactive temperature-based MRI reconstruction for real-time diagnostics and therapy.

## Figures and Tables

**Table 1:** Mean ± standard deviation SSIM, NRMSE, and PSNR values obtained using the state-of-the-art methods with different AFs.

AFs	Methods	Type	SSIM	NRMSE	PSNR
	Zero-filling		0.654 ± 0.04	0.038 ± 0.08	23.93 ± 2.62
	dAUTOMAP	DM	0.849 ± 0.04	0.025 ± 0.02	27.87 ± 1.93
	Unet	SD	0.977 ± 0.06	0.023 ± 0.01	33.28 ± 3.14
	DAGAN	SD	0.963 ± 0.10	0.029 ± 0.02	31.33 ± 3.11
	RefineGAN	SD	0.979 ± 0.07	0.019 ± 0.01	35.44 ± 3.71
	PBCU	SD	0.983 ± 0.06	0.018 ± 0.03	34.69 ± 3.97
4×	FDA-CNN	SD	0.981 ± 0.05	0.012 ± 0.01	38.86 ± 4.05
	DC-CNN	DD	0.986 ± 0.05	0.012 ± 0.01	39.51 ± 3.35
	KIKI-net	DD	0.986 ± 0.06	0.012 ± 0.01	39.64 ± 3.35
	W-net	DD	0.985 ± 0.06	0.014 ± 0.01	38.23 ± 3.32
	Hybrid cascade	DD	0.986 ± 0.03	0.012 ± 0.01	39.87 ± 3.38
	Dual-encoder Unet	DD	0.980 ± 0.05	0.018 ± 0.02	34.53 ± 1.35
	RA-CNN	DD	0.989 ± 0.03	0.013 ± 0.00	41.95 ± 3.21
	Zero-filling		0.593 ± 0.05	0.055 ± 0.01	23.63 ± 2.67
	dAUTOMAP	DM	0.823 ± 0.02	0.051 ± 0.25	27.20 ± 1.51
	Unet	SD	0.966 ± 0.10	0.027 ± 0.02	31.88 ± 3.13
	DAGAN	SD	0.949 ± 0.11	0.039 ± 0.01	28.69 ± 2.66
	RefineGAN	SD	0.973 ± 0.08	0.023 ± 0.01	33.84 ± 3.83
	PBCU	SD	0.983 ± 0.06	0.021 ± 0.02	33.25 ± 2.76
5×	FDA-CNN	SD	0.976 ± 0.05	0.016 ± 0.01	33.31 ± 4.06
	DC-CNN	DD	0.982 ± 0.07	0.015 ± 0.01	37.67 ± 3.20
	KIKI-net	DD	0.982 ± 0.07	0.015 ± 0.02	37.67 ± 3.22
	W-net	DD	0.981 ± 0.06	0.017 ± 0.01	36.50 ± 3.21
	Hybrid cascade	DD	0.982 ± 0.08	0.014 ± 0.02	37.88 ± 3.25
	Dual-encoder Unet	DD	0.975 ± 0.04	0.025 ± 0.03	33.24 ± 1.70
	RA-CNN	DD	0.986 ± 0.04	0.015 ± 0.01	41.11 ± 3.23

DM: direct mapping; SD: single-domain network; DD: dual-domain network. dAUTOMAP: decomposing automated transform by manifold approximation; DAGAN: de-aliasing generative adversarial network; PBCU: projection-based cascade Unet; CNN: convolutional neural network; FDA-CNN: fully dense attention CNN; DC-CNN: deep cascade CNN; RA-CNN: residual attention CNN; AF: acceleration factor; SSIM: structural similarity index measure; NRMSE: normalized root mean square error; PSNR: peak signal-to-noise ratio.

**Table 2:** Mean ± standard deviation SSIM, NRMSE, and PSNR values of the state-of-the-art methods obtained using AF 6.

Methods	SSIM	NRMSE	PSNR	Parameters (M)
Zero-filling	0.651 ± 0.09	0.071 ± 0.08	20.63 ± 3.15	-
dAutomap	0.692 ± 0.09	0.033 ± 0.03	24.73 ± 3.01	0.16
Unet	0.701 ± 0.09	0.031 ± 0.02	27.43 ± 2.49	3.13
PBCU	0.801 ± 0.05	0.035 ± 0.07	31.25 ± 1.43	3.15
FDA-CNN	0.816 ± 0.18	0.022 ± 0.02	31.88 ± 4.00	1.01
DC-CNN	0.779 ± 0.12	0.030 ± 0.03	30.31 ± 3.40	1.39
KIKI-net	0.756 ± 0.05	0.026 ± 0.09	30.93 ± 1.49	1.25
W-net	0.731 ± 0.07	0.038 ± 0.02	30.70 ± 3.66	1.13
Hybrid cascade	0.813 ± 0.11	0.025 ± 0.12	31.19 ± 2.40	1.66
Dual-encoder Unet	0.751 ± 0.08	0.024 ± 0.02	25.68 ± 3.00	0.99
RA-CNN	0.848 ± 0.19	0.021 ± 0.04	37.30 ± 4.04	0.68

M = million.

## Data Availability

The dataset is accessible from: https://sites.google.com/view/calgary-campinas-dataset/home (accessed on 20 January 2023). The source code of this manuscript is available at https://github.com/biddut2j8/RA-CNN (last accessed on 20 March 2023).
